# A patient with aortic annular rupture secondary to blood culture–negative endocarditis: A case report

**DOI:** 10.3389/fcvm.2026.1797434

**Published:** 2026-03-30

**Authors:** Mingjiao Hua, Junjie Sun, Yahong Wang, Xiaohang Wan, Can Liu, Lihong Wang

**Affiliations:** 1 School of Medical Imaging, Binzhou Medical University, Yantai, Shandong, China; 2 Department of Ultrasound, Qingdao University Medical College Affiliated Yantai Yuhuangding Hospital, Yantai, Shandong, China; 3 School of Medical Imaging, Shandong Second Medical University, Weifang, Shandong, China; 4 Department of Ultrasound, Binzhou Medical University Hospital, Yantai, Shandong, China

**Keywords:** aortic annular rupture, blood culture–negative infective endocarditis, second-generation gene sequencing, transesophageal echocardiography, unicuspid aortic valve

## Abstract

We report a rare case of aortic valve annular rupture secondary to culture-negative infective endocarditis. This report aims to summarize the diagnostic challenges, imaging-based differentiation, and microbiological strategies, serving as a reference for similar clinical scenarios. The patient presented with progressively worsening palpitations and chest tightness. Initial transthoracic echocardiography (TTE) suggested a unicuspid aortic valve, but subsequent transesophageal echocardiography (TEE) revealed an aortic valve annular rupture with severe regurgitation. The patient exhibited signs of infection, but both blood cultures and sputum bacteriology tests were negative. Surgical exploration revealed infective changes in the valve annulus; combined with valve pathology and second-generation gene sequencing results, the diagnosis of culture-negative infective endocarditis was confirmed according to the 2023 Duke-ISCVID (Duke-International Society of Cardiovascular Infectious Diseases) criteria. This case posed diagnostic challenges due to the transthoracic echocardiographic appearance mimicking a unicuspid aortic valve and the negative routine microbiological tests. It underscores the importance of promptly performing transesophageal echocardiography when encountering suspected rare valvular abnormalities. For suspected infective endocarditis (IE) with negative routine microbiological tests, early tissue molecular sequencing should be performed to clarify the pathogen.

## Introduction

The aortic annulus is part of the cardiac fibrous skeleton, connecting the left ventricle and the aortic root. On one side, it interfaces with the aortic valve, and on the other, with the interventricular septum, the anterior mitral leaflet, and the free wall of the left ventricle. Aortic annular rupture can be classified as suprannular, annular, subannular, or mixed. Its clinical manifestations closely correlate with the rupture type (1). Contained ruptures usually do not require emergency cardiac surgery, whereas uncontained ruptures often necessitate emergent surgical intervention, with in-hospital mortality rates as high as 75% (2).

## Case description

**Figure 1:**
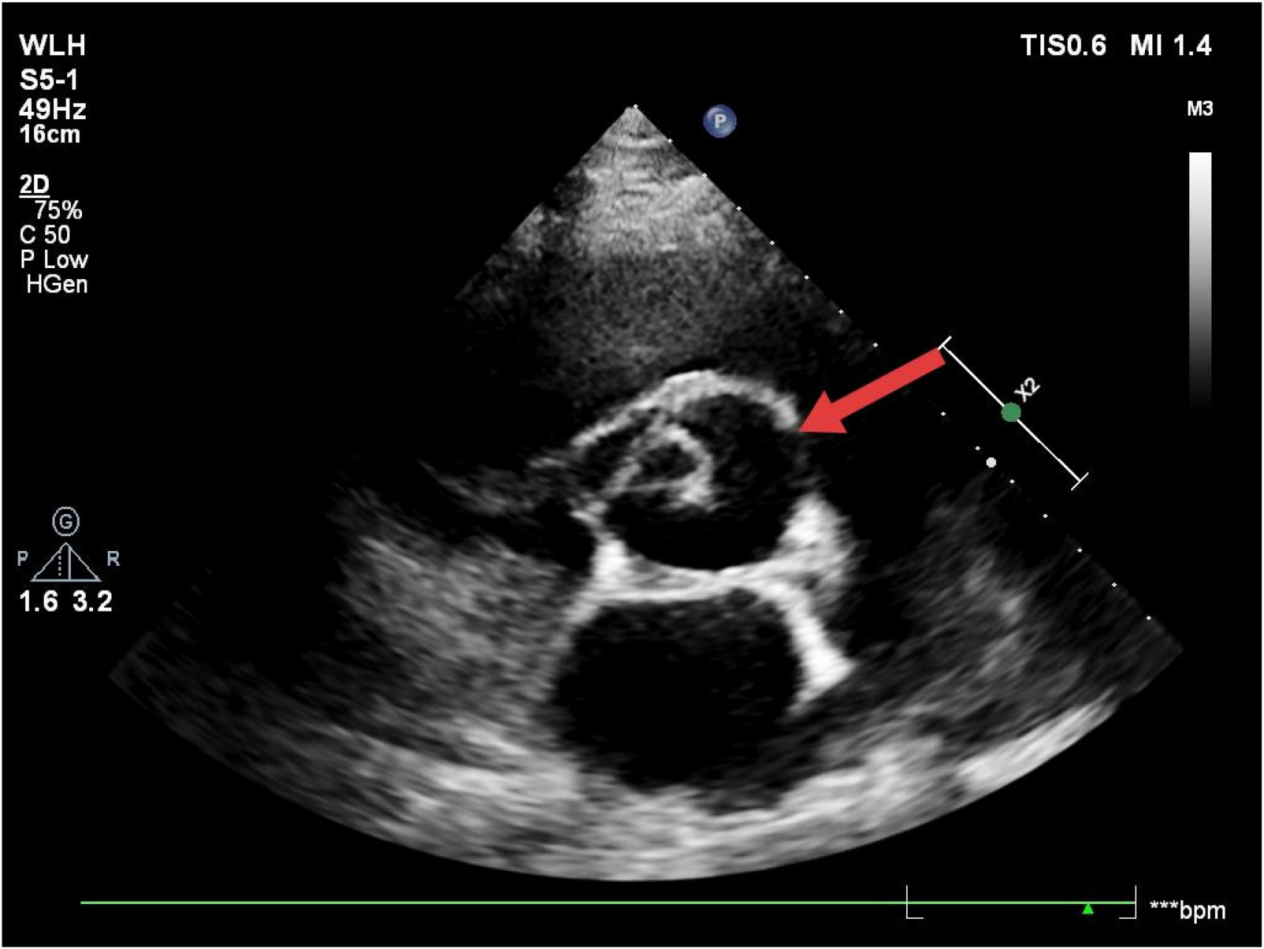
Transthoracic echocardiography (short-axis view of the great arteries). The arrow indicates the aortic valve, which appears to be a unicuspid valve.

**Figure 2:**
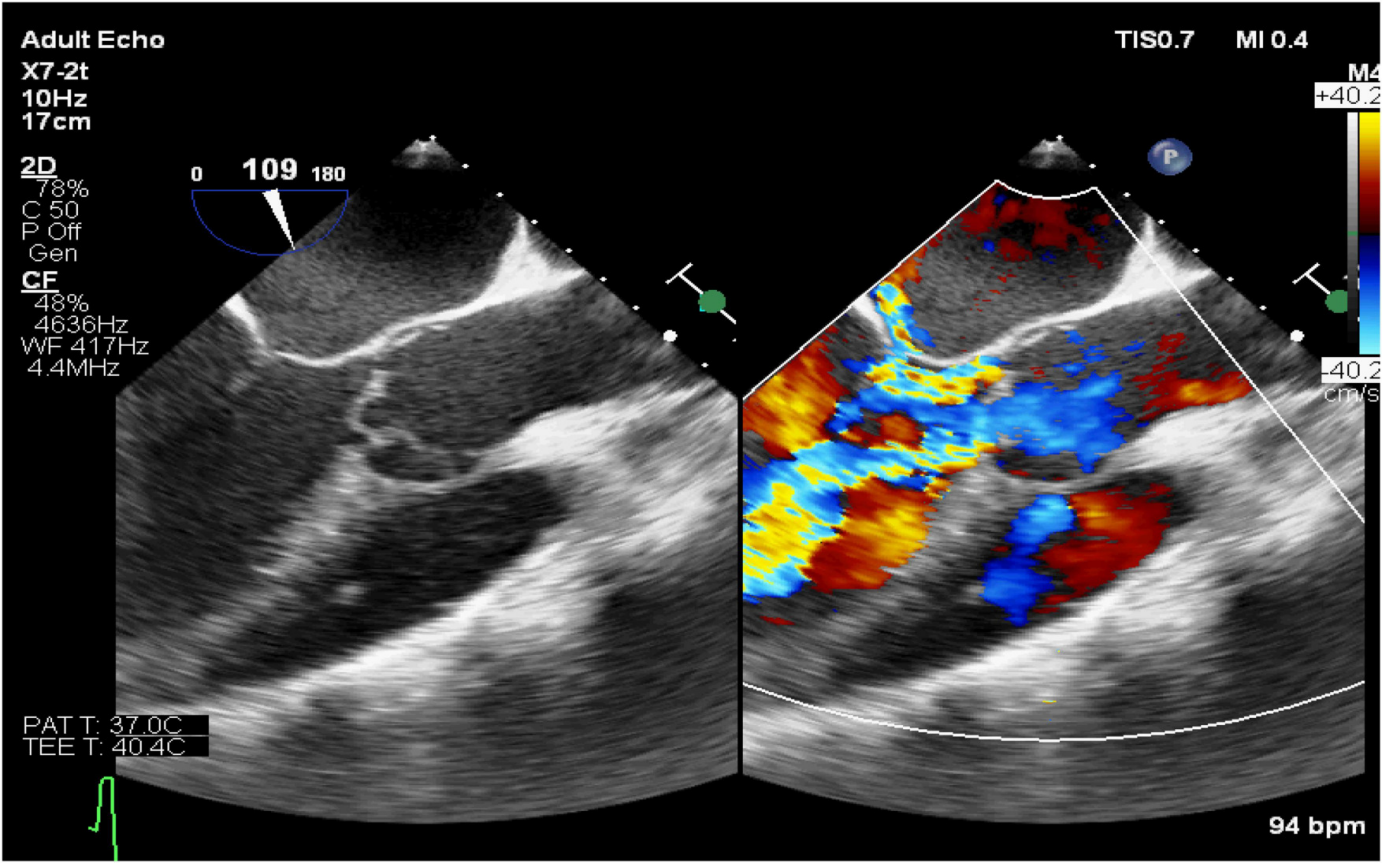
Transesophageal echocardiography (long-axis view of the left ventricle). Left panel: Rupture and detachment of the left and right coronary valve annuli, with the valve leaflets swinging along with the annuli. Right panel: Severe aortic valve regurgitation is observed.

**Figure 3:**
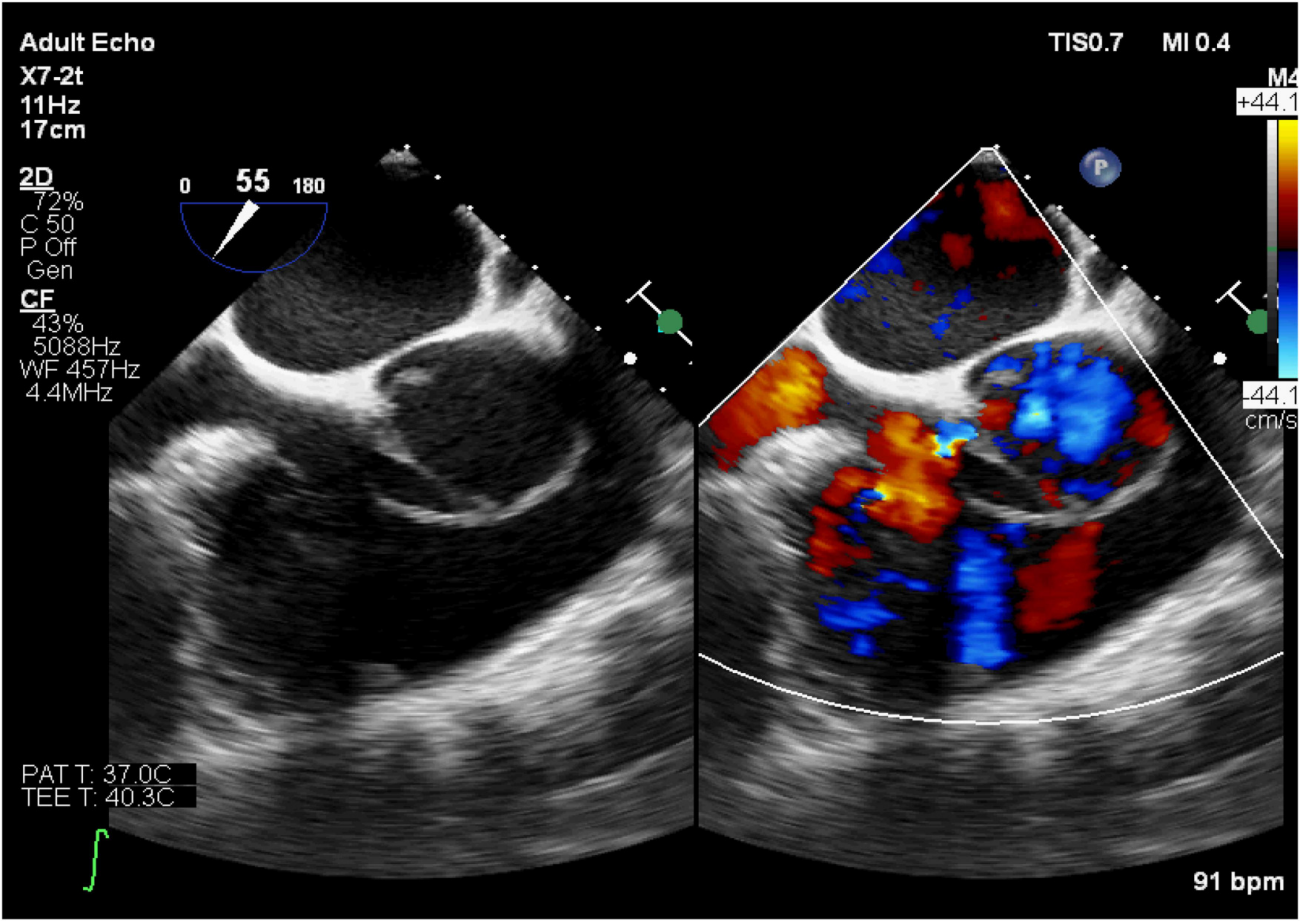
Transesophageal echocardiography (short-axis view of the aortic valve). Left panel: The aortic valve appears tricuspid, with the non-coronary cusp still attached to the annulus; rupture and detachment of the left and right coronary valve annuli are present, with the valve leaflets swinging along with the annuli. Right panel: Severe aortic valve regurgitation is observed.

The patient is a 46-year-old male working in machine manufacturing with a history of exposure to metal dust. He has a previous history of cerebral infarction but no other past medical history. Six months prior to presentation, the patient developed palpitations and chest tightness without an obvious trigger. Computed tomography revealed pleural effusion, and symptoms improved after diuretic therapy. Four days before admission, his symptoms worsened markedly, prompting transfer to our hospital. Physical examination revealed a grade 2/6 systolic murmur at the cardiac apex and a grade 5/6 diastolic murmur over the aortic valve area. Blood test results were as follows: C-reactive protein (CRP) 16.3 mg/L, white blood cell count (WBCC) 11.4 × 10^9^/L, absolute neutrophil count (ANC) 8.13 × 10/L, brain natriuretic peptide (BNP) 458 pg/mL, high-sensitivity cardiac troponin I (hs-cTnI) 127.3 pg/mL, erythrocyte sedimentation rate (ESR) 21 mm/h, serum creatinine (CREA) 80 μmol/L. Initial transthoracic echocardiography (TTE) suggested a unicuspid aortic valve (UAV) with severe regurgitation (Figure 1). However, isolated aortic stenosis (AS) is the predominant lesion in unicuspid aortic valve disease, whereas isolated aortic regurgitation (AR) is relatively uncommon (3–5). Moreover, transesophageal echocardiography (TEE) is more accurate than TTE for diagnosing a unicuspid aortic valve (6, 7). TEE was subsequently performed to better define the valvular anatomy. The examination ruled out the initial diagnosis and demonstrated aortic annular rupture with valve prolapse and severe regurgitation (Figures 2, 3). Based on the Echocardiographic images, rupture and detachment of the left and right coronary valve annuli were observed, with the valve leaflets moving along with the annuli. Vena contracta width (VCW) was significantly increased (>0.6 cm), the effective regurgitant area (ERA) was markedly enlarged (>0.3 cm^2^), and enlargement of the left atrium and left ventricle was noted. Following the exclusion of other diagnoses and a comprehensive multi-parameter evaluation, the final diagnosis was confirmed as “rupture of the aortic valve annulus with valve prolapse and severe regurgitation” (Figures 2, 3). This finding drew attention to a rare pathology. Upon admission, the patient was immediately treated with symptomatic supportive medications, including positive inotropes, diuretics, myocardial nutrients, and heart function enhancers. Given intermittent fever (up to 38 °C), along with elevated white blood cell and neutrophil counts, piperacillin-tazobactam was administered for one week. Following treatment, the patient's temperature normalized, white blood cell and neutrophil counts returned to normal, and infection symptoms were effectively controlled. Infective endocarditis was initially suspected during the etiological investigation of the annular rupture. However, blood cultures and sputum bacteriological examinations yielded no microbiological evidence of infection. Review of the patient's medical history identified recurrent oral ulcers, raising the possibility of immune-mediated conditions such as Behçet's disease. A consultation with rheumatology and immunology specialists was requested. Further examination revealed that rheumatoid factor, autoantibodies, and other relevant markers were within normal limits, and the results did not support the diagnosis.

**Figure 4:**
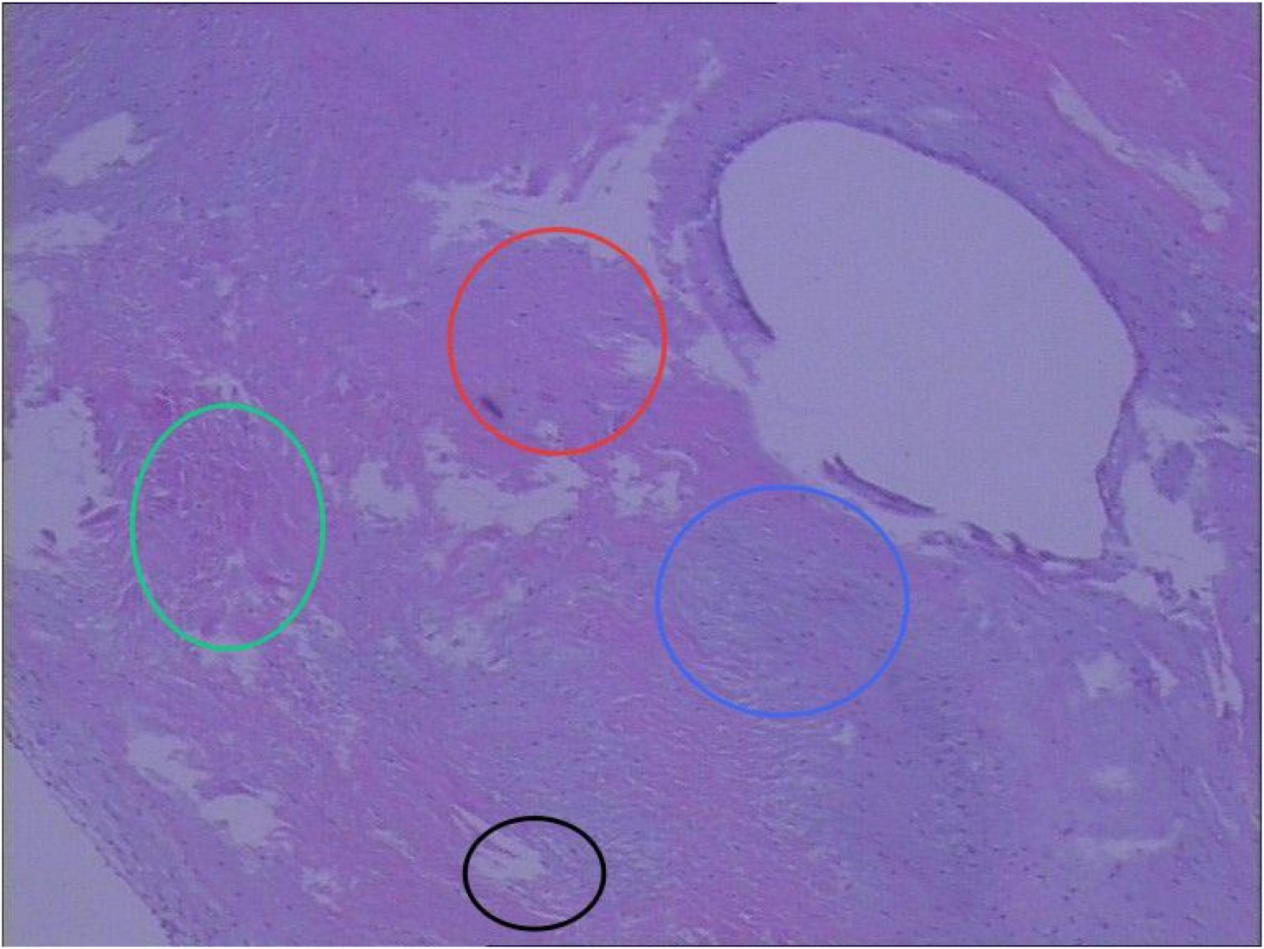
Hematoxylin and eosin (HE) staining of the excised valve tissue showed that the red, green, blue, and black circles corresponded to fibrous tissue hyperplasia, hyaline degeneration, myxoid degeneration, and focal necrosis, respectively.

Conservative medical management was ineffective. Surgical intervention was performed after infection control and clinical stabilization. Intraoperatively, localized millet-seed–like changes were observed in the aortic annulus and the right coronary cusp, suggesting a possible infectious etiology. Histopathological examination of the excised valve tissue revealed fibrous tissue hyperplasia with hyaline and myxoid degeneration and focal necrosis (Figure 4). The above histopathological changes suggest that the valve tissue has been subjected to inflammatory invasion, leading to structural integrity disruption, providing important histological evidence for infectious damage. Especially in the absence of positive blood culture results, pathological evidence became one of the key pieces of evidence for the diagnosis of “infective endocarditis.” Combined with the intraoperative signs of infection and the results of second-generation gene sequencing (Staphylococcus hominis, cytomegalovirus), the diagnosis of infective endocarditis was confirmed.

## Discussion

According to the 2023 updated Duke-ISCVID diagnostic criteria for infective endocarditis (8), the patient meets the key diagnostic points for “definitive infective endocarditis”: 1. The infectious lesions observed during surgery meet the newly added Surgical major criteria. 2. Second-generation sequencing of the valve tissue detected Staphylococcus hominis, fulfilling the major microbiological criterion (this criterion now includes molecular diagnostic technologies such as PCR and metagenomic sequencing for pathogen detection). 3. The patient exhibited intermittent fever, elevated white blood cell count, and other minor clinical manifestations. The application of this diagnostic framework helped clarify the underlying cause of the aortic valve annular rupture in this case. Notably, upon admission, the patient, due to the presence of fever and elevated white blood cell count suggesting infection, was immediately started on empirical antibiotic therapy with piperacillin-tazobactam. The use of antibiotics suppressed and cleared the pathogenic bacteria from peripheral blood and respiratory secretions, which reduced the number of pathogenic microorganisms. This became the main reason for the negative blood cultures and sputum bacteriology tests. Additionally, Staphylococcus hominis is less virulent than other common pathogens of infective endocarditis (9, 10) and has a slower replication rate, which increased the difficulty of clinical microbiological diagnosis. Therefore, when both blood culture and sputum bacteriology results are negative, but other clinical manifestations meet the IE diagnostic criteria, clinicians should be alert to the possibility of culture-negative infective endocarditis due to factors such as antibiotic use and the characteristics of the pathogenic microorganism.

Aortic annular rupture is a rare and life-threatening complication. Timely recognition, intervention, and identification of the underlying etiology are critical for a favorable outcome. In addition to the etiology involved in this case, this complication may also occur during transcatheter aortic valve replacement (11).

The direct echocardiographic signs of aortic valve annulus rupture include separation between the aortic valve annulus attachment site and the cardiac tissue, with abnormal blood flow signals detectable in the separated area on color Doppler. This is accompanied by abnormal leaflet motion with significantly increased amplitude. After the rupture of the aortic valve annulus in this case, the valve lost the support of the fibrous ring, leading to prolapse, displacement, and abnormal motion. The normal coaptation structure between the leaflets was disrupted. Following rupture, the annulus and valve underwent morphological and structural disarray due to the injury. On transthoracic echocardiography (TTE), the typical “Y”-shaped closure of the normal tricuspid valve was not visible, and the characteristic morphology of a bicuspid valve was also not seen. Instead, only a mass of abnormal oscillating fibrous tissue was detected. This appearance is similar to that of a unicuspid aortic valve, which was an important pathological basis for the initial misdiagnosis. Moreover, TTE is the initial screening tool for cardiac ultrasound, but it is limited by chest wall, lung, and rib obstruction, and its spatial resolution of structures like the aortic root and valve annulus is insufficient. As a result, it is unable to clearly distinguish the continuity of the annulus or the fine structure of the perivalvular tissue. The limitations of this technique further increased the difficulty of differential diagnosis and highlighted the necessity of promptly using transesophageal echocardiography (TEE) in cases of suspected complex valvular lesions.

On transthoracic echocardiography, unicuspid aortic valve exhibits characteristic features, including a single commissural attachment, a circular leaflet-free edge opposite the commissure, and an eccentric systolic orifice (12). These findings help distinguish a unicuspid aortic valve from other conditions during echocardiographic assessment.

In addition to the conditions mentioned above, echocardiographic evaluation should distinguish these from other diseases to avoid misdiagnosis. One such condition is congenital bicuspid aortic valve (BAV) with leaflet prolapse, which often presents a characteristic “fish-mouth” orifice (13) on parasternal short-axis views and typically follows a chronic course, whereas aortic annular rupture usually presents acutely. Cardiac involvement in Behçet's disease can also produce echocardiographic findings similar to the perivalvular lesions observed in infective endocarditis (14). Although rare in China (15), this possibility was considered in the present case and was ultimately excluded based on clinical history and laboratory findings.

## Conclusion

This case describes a rare presentation of aortic annular rupture associated with blood culture–negative infective endocarditis, involving a complex diagnostic and therapeutic process. The patient was initially suspected of having a unicuspid aortic valve based on transthoracic echocardiography, while subsequent transesophageal echocardiography established the diagnosis of annular rupture. Immune-mediated diseases were excluded, and the infectious etiology was confirmed by surgical findings, pathological evaluation, and next-generation sequencing. This case highlights that when transthoracic echocardiography suggests a rare valvular malformation, transesophageal echocardiography should be promptly performed for accurate differentiation. Furthermore, when investigating the etiology of annular rupture, if routine microbiological cultures are negative but clinical features suggest infective endocarditis, blood culture–negative infective endocarditis should be considered. This case provides practical insights into the diagnosis and management of similar rare clinical presentations.

## Data Availability

The original contributions presented in the study are included in the article/Supplementary Material, further inquiries can be directed to the corresponding author.
